# Barriers to Health Care in Rural Mozambique: A Rapid Ethnographic Assessment of Planned Mobile Health Clinics for ART

**DOI:** 10.9745/GHSP-D-14-00145

**Published:** 2015-03-02

**Authors:** Amee Schwitters, Philip Lederer, Leah Zilversmit, Paula Samo Gudo, Isaias Ramiro, Luisa Cumba, Epifanio Mahagaja, Kebba Jobarteh

**Affiliations:** a Centers for Disease Control and Prevention, Center for Global Health, Atlanta, GA, USA; b Centers for Disease Control and Prevention, Epidemic Intelligence Service, Atlanta, GA, USA; c Centers for Disease Control and Prevention, Maputo, Mozambique; d Association of Schools and Programs of Public Health, Washington, DC, USA; e Mozambique Ministry of Health, Xai Xai, Mozambique; f Ministry of Health, Zambezia, Mozambique

## Abstract

**Background:**

In Mozambique, 1.6 million people are living with HIV, and over 60% of the population lives in rural areas lacking access to health services. Mobile health clinics, implemented in 2013 in 2 provinces, are beginning to offer antiretroviral therapy (ART) and basic primary care services. Prior to introduction of the mobile health clinics in the communities, we performed a rapid ethnographic assessment to understand barriers to accessing HIV care and treatment services and acceptability and potential use of the mobile health clinics as an alternative means of service delivery.

**Methods:**

We conducted assessments in Gaza province in January 2013 and in Zambezia Province in April–May 2013 in districts where mobile health clinic implementation was planned. Community leaders served as key informants, and chain-referral sampling was used to recruit participants. Interviews were conducted with community leaders, health care providers, traditional healers, national health system patients, and traditional healer patients. Interviewees were asked about barriers to health services and about mobile health clinic acceptance.

**Results:**

In-depth interviews were conducted with 117 participants (Gaza province, n = 57; Zambezia Province, n = 60). Barriers to accessing health services included transportation and distance-related issues (reliability, cost, and travel time). Participants reported concurrent use of traditional and national health systems. The decision to use a particular health system depended on illness type, service distance, and lack of confidence in the national health system. Overall, participants were receptive to using mobile health clinics for their health care and ability to increase access to ART. Hesitations concerning mobile health clinics included potentially long wait times due to high patient loads. Participants emphasized the importance of regular and published visit schedules and inclusion of community members in planning mobile health clinic services.

**Conclusion:**

Mobile health clinics can address many barriers to uptake of HIV services, particularly related to transportation issues. Involvement of community leaders, providers, traditional healers, and patients, as well as regularly scheduled mobile clinic visits, are critical to successful service delivery implementation in rural areas.

## Abstract (precis)

Mobile health clinics can markedly decrease clients' transportation time and cost to access antiretroviral therapy (ART) and other health services in rural areas, potentially improving use. Close coordination with community leaders and regularly scheduled visits by the mobile clinics are critical.

## INTRODUCTION

Globally, the annual numbers of new HIV infections and AIDS deaths are declining.^1^ However, sub-Saharan Africa continues to bear the greatest HIV burden, with 70% of all cases occurring in the region. As of 2012, estimates indicate that only half of people living with HIV (PLHIV) in sub-Saharan Africa knew their HIV status.^1^ While much progress has been made, and 54% of persons eligible for antiretroviral therapy (ART) in low- and middle-income countries are now receiving it, almost half remain without access. Health systems in the region face the difficulty of increasing the number of patients on ART while simultaneously caring for millions of people already on ART due in part to the critical shortage of health facilities and health care workers.^2^

Almost half of people in sub-Saharan Africa who are eligible for ART don't have access to it.

Mozambique is one of the countries most affected by HIV/AIDS worldwide. The HIV epidemic is generalized with an estimated prevalence of 11.5%, although regional variations exist. Gaza province, for example, has the highest prevalence in the country at 25.1%.^3^ Increasing ART coverage among the estimated 1.6 million PLHIV in the country remains a major challenge as over 60% of the population lives in rural areas and lacks access to health services.^3^ ART coverage is low in key provinces throughout the country. For instance, Zambezia, Gaza, Sofala, and Manica provinces account for 52% of all PLHIV (20%, 12%, 11%, and 9%, respectively) but only 40% of the people currently receiving treatment in the country.^4^ Targeted ART scale-up in these provinces will increase national treatment coverage.

Over 60% of Mozambique's population lives in rural areas with limited access to health services.

Mozambique is one of the world's poorest countries—185th of 187 countries in level of development according to the United Nations Development Index.^5^ Mozambique's severe shortage of health facilities means patients must routinely travel long distances to access health care. In addition, stigma causes unnecessary suffering among PLHIV and has been cited as a barrier to HIV care and treatment services.^6^^–^^8^ Another potential barrier to HIV care and treatment is the reliance on traditional healers,^9^ which people use for a variety of reasons including easier access, confidence in their treatments, and local cultural practices.^10^^,^^11^

Mobile health clinics are a potential solution to some of these barriers. Mobile health clinics are specially designed four-wheel drive trucks that are able to reach isolated destinations and are equipped to provide comprehensive health services. Services are provided in the vehicle as well as in tents or other structures nearby. Such mobile health clinics providing limited HIV, tuberculosis (TB), malaria, and sexually transmitted infection (STI) services have been successfully piloted in neighboring Malawi and South Africa.^12^^,^^13^

Mobile health clinics are special off-road vehicles capable of providing health services in rural areas.

In 2013, the Ministério de Saúde (MISAU), with support through the US President's Emergency Plan for AIDS Relief (PEPFAR), implemented mobile health clinics in rural districts of Mozambique.^14^ The objectives of the program are to scale-up HIV care and treatment services in areas with low treatment coverage, improve ART retention, provide integrated TB/HIV services, and provide selected primary health and maternal and child health services. By design, mobile health clinics address geographic and environmental access issues. However, the success of mobile health clinics in rural Mozambique depends on whether community members will accept and use them. Thus, a study was conducted prior to implementation of the mobile health clinics to: (1) establish current community health practices and attitudes, including potential acceptance and use of mobile health clinics, and (2) provide a baseline understanding of the health care seeking practices in high-burden HIV/AIDS rural communities in 2 provinces where mobile health clinics were to be introduced.

## METHODS

We conducted a cross-sectional, rapid ethnographic evaluation in January and April–May 2013. This approach facilitates the collection of locally relevant data from the perspective of those most affected by the research question(s).^15^ The process is inclusive of a triangulation method of structured interviews, semi-structured interviews, and observation. These modified procedures allow for relatively rapid research to enhance and guide program planning and evaluation and have been demonstrated to be a successful adaptation of traditional ethnographic research methods.^16^

Selected districts within Gaza and Zambezia provinces that were going to receive mobile health clinic services were included in the assessment because of their population size and current ART coverage. Within these districts, specific towns were selected for their representativeness of communities that would be receiving mobile health care services. Representation included availability of health care services and health care providers in the community, distance to services, and population size. In addition, the communities in Zambezia were chosen by matching population size and proximity to the nearest ART health facility to the communities that were chosen in Gaza. Approval for the assessment was obtained from the Centers for Disease Control and Prevention and from the Mozambique Ministry of Health.

### Recruitment of Participants

In January 2013, a total of 57 participants (n = 39 women, n = 18 men) were interviewed in Gaza province. Gaza province is located in southern Mozambique, immediately north of the capital city of Maputo. The 57 interviews were conducted in 2 districts in Gaza province: Chibuto and Mandlakaze.

From April to May 2013, a total of 60 participants (n = 17 women, n = 43 men) were interviewed in Zambezia province, located in northern Mozambique and bordering Malawi. The 60 interviews were conducted in 2 districts in Zambezia province: Morrumbala and Milange.

Participants and sampling locations were purposively selected in close collaboration with the MISAU and PEPFAR implementing partners that provide HIV care and treatment services in the selected districts, including the Elizabeth Glaser Pediatric AIDS Foundation, the International Center for AIDS Care and Treatment Programs, and Friends in Global Health. Prior to arriving in the community, the implementing partners verbally communicated the purpose of the study to community leaders and sought permission to conduct interviews in the community.

Community leaders served as key informants, and chain-referral sampling was used to recruit participants. Community leaders were asked to help identify people in their communities where mobile health clinics were planned. Community leaders were also asked to help identify traditional healers who were willing to be interviewed, and, in turn, traditional healers were asked to identify patients willing to be interviewed. Hospital/clinic staff were informed of the assessment and asked to participate in the interviews and to help identify community members accessing national health services who were willing to be interviewed.

We conducted qualitative interviews with each of 5 populations: national health system providers (doctors, medical technicians, nurses, and lay counselors), persons accessing health care, traditional healers, persons accessing care from traditional healers, and community leaders. All participants were at least 18 years old and provided consent to be interviewed.

Interviews were conducted with community leaders, national health system providers and their patients, and traditional healers and their patients.

### Data Collection

Audio-recorded interviews, lasting 0.5 to 2 hours, were conducted in either Portuguese or a local language (Shangana or Chope in Gaza, Chichewa or Lolo in Zambezia) and translated into English. Site visit teams were comprised of 2 primary investigators from CDC Atlanta, a Mozambique-assigned fellow from the Association of Schools and Programs of Public Health, and between 2 and 3 persons from local implementing partners. Interviews were conducted through a translator or, when possible, through one of the primary investigators.

Before beginning the interviews, the interviewer read each participant an informed consent form and obtained written consent to be interviewed. No participant declined participation or rescinded informed consent during the study. No compensation was provided for participation. After transferring the audio recordings to a computer and verifying transfer, the original recordings were deleted. The transferred recordings were stored on a password-protected computer. Consent forms and de-identified transcripts were stored in separate locked file cabinets belonging to the primary investigators.

### Data Analysis

All coding and thematic analyses were conducted manually. The data analysis process included reading, coding, summarizing, and interpreting, leading to identification of common themes.

## RESULTS

Slightly more than half (52%) of the participants in the interviews in both Gaza and Zambezia combined were men (Table). Among the participants who were patients (traditional healer patients and national health system patients), 48% were women, while all community leaders were males (n = 13). Most (66%) health care providers (both traditional healers and providers from the national health system) were women. The average age of male participants was 47.4 years (range, 19–81 years), slightly older than the average age of female participants, which was 38.6 years (range, 22–65 years).

**Table:** TABLE. Number of Interviewees by Type and Sex, in Gaza and Zambezia Provinces of Mozambique

	**Gaza**		**Zambezia**		**TOTAL**
	**Men**	**Women**	**Men**	**Women**	
Community Leaders	5	0	8	0	13
NHS Providers	3	7	4	2	16
Traditional Healers	3	9	2	5	19
NHS Patients	6	14	19	10	49
Traditional Healer Patients	1	9	10	0	20
TOTAL	18	39	43	17	117

Abbreviation: NHS, national health system.

The interviews explored the following themes: community and health problems; barriers to health care; and knowledge and attitudes about mobile health clinics.

### Community and Health Problems

Participants were asked to self-identify the 3 problems they perceived to have the biggest impact on their community, their personal health, and their family's overall health. The most commonly cited community problems were lack of schools, lack of access to health care services, and lack of food (hunger). In Zambezia, many participants (n = 26) also cited lack of clean water (compared with 6 participants in Gaza). Because of the lack of access to health care services, people frequently delayed going to health facilities for minor health problems. The most commonly cited health problems were malaria, HIV, diarrhea, and TB (in order of frequency reported).

Commonly cited community problems included lack of schools, food, clean water, and access to health care.

Lack of access to health services often had impacts beyond individual and family health. While the costs associated with obtaining medical care once at the facility are minimal, getting to the facility in the first place requires a large sum of money that is much needed for food, housing- and school-related costs, and other supplies. One patient stated:

They [health problems] have a negative impact because my family has to use money for transportation to hospital for medication. If I didn't have HIV, we could use that money for other things.

In Gaza, lack of health services was the third most mentioned community problem behind lack of schools and lack of food. In Zambezia, lack of health facilities was also mentioned, but lack of water, food, schools, and transportation, and the poor condition of roads, were all mentioned more often. Beyond financial costs, time-associated costs with accessing health care were described as sometimes prohibitive, with round trips to health care facilities taking more than 24 hours in some areas. One patient described the impact of not having a health facility in the community:

Access to health care is a big problem. I left my home this morning to reach here. We have a small clinic, but they don't do blood tests. Sometimes we have transport, but today because of the rain it was difficult. It takes about 3 hours to walk, more if you are sick.

### Barriers to Health Care

The most commonly cited barriers to health care were distance from health facilities, lack of money, lack of available transportation, and poor health facility conditions. Distance was seen as a barrier to accessing health care services because of both the cost and the lack of transportation. Participants reported that it took them an average of 1 hour in Gaza and 3 hours in Zambezia to reach a health facility. Gaza participants stated that minibus taxis were used as their primary means of transportation. In Zambezia, few participants had access to minibuses and described arriving to clinics on foot or by bicycle. Participants in Milange district in Zambezia commonly used a boat to cross a crocodile-infested river in order to travel to the nearest health facility, which often meant going to Malawi. Many of the patients felt that the distance affected their ability to receive services.

Distance, cost, and lack of transportation were commonly cited barriers to health care access.

One patient in Gaza explained:

*People feel the services are at fault. People get up at 3 am to catch the chapa [minibus]* *to get here and wait for a consult, they go home about 4 pm. Most people have children, when they go to the hospital they have no person to give food or assistance to the children.*

In Milange district (Zambezia province), people faced especially grueling journeys to reach the HIV clinic. One provider described the barriers his patients overcame to access care:

People bicycle all day to come to the ART clinic—they start very early in the morning, travel 150 km, arrive very late at night, sleep in a family member's house, and then the next day come to the clinic for treatment.

Stock-outs combined with the long distances and high costs associated with reaching health care facilities in rural areas can negatively impact an individual's ability to remain adherent to their medications. This can ultimately have severe consequences for the individual. A patient explained the challenge of poverty in rural Mozambique and the inability to easily and/or frequently access health care:

My big concern is that I came here early in the morning to get pills for my son, he is sick. They [health care workers] only gave me one kind because they are out of the other. I don't have money to go to the hospital to get the pills. They tell me to come back tomorrow or next week to get the pills, but I don't have the money and it takes time to come here.

Distance and high costs associated with accessing health facilities can negatively impact medication adherence.

Many patients stated that they concurrently used traditional healers or would switch health systems if they were not feeling better after visiting one of the health systems. Traditional and formal health system treatments were described as being considerably different for some diseases; however, all interviewed patients stated that traditional healers had referred them to the formal health system for HIV treatment. The reasons for the use of traditional healers depended on the nature of the illness. One traditional healer patient described the concurrent use of traditional healers and the national health system:

You get sick, you go to the hospital and you get worse because you have a lack of blood and water in the body, and you get worse, so you go to traditional healer.

Other patients described their use of traditional healers when they could not reach a health facility. One traditional healer patient described her experience:

*I couldn't go to the hospital, and I was having severe leg pain. The health center was far. I had no car, I had no phone. I crawled on my knees to the traditional healer's house. I got there at 1 am, when I got there I knocked, but no one answered, so I slept outside. The next day he treated me. I had xifulu.* [*Xifulu was described as bad spirits sent from one person to another.]*

### Potential Acceptance of Mobile Health Clinics

All participants supported the idea of mobile health clinics, stating they would use the clinics if available in their communities. One patient commented:

We would be proud to use the mobile clinics. Our children, our future, could receive treatment close to home. The only potential obstacle would be if the medications were expensive.

A national health system provider explained the potential benefit of offering multiple services at the mobile health clinics:

They will be good services if they really come to help. I would love to have services for the youngest, especially girls. If we see girls here that are just starting to look like women, they often come for contraception, but older people won't let the girls get contraception. It is thought that if a girl is looking for contraception, she is a prostitute. If anyone sees a girl this age at the clinic by herself, they will tell her parents, because they assume it is for contraception. Young girls are pregnant here, but we are not allowed to give contraception.

While universally supported, distance and money were potential barriers to using the mobile health clinics. One patient explained:

I live alone, and I work on a farm. It takes me 2 hours to get to the clinic, and costs 80 meticais [about US$2.41] round trip. I'd like to use the mobile clinics. The only barrier would be if they cost money and I didn't have it.

Participants explained that successful mobile health clinic implementation would depend on coordination with community leaders and traditional healers. They reported that community leader support was an important factor in use of the mobile health clinics.

Coordination with community leaders and traditional healers is crucial for successful mobile health clinic implementation.

In addition, after implementation of mobile health clinics, the community asked that they be kept informed of scheduled visit days and potential deviations from these days. There was concern regarding a lack of notification over scheduled visit dates and a fear of not being able to be seen because of the volume of people using the clinics. One patient of a traditional healer explained:

A [vaccination] clinic came to Chaimite last week but not all of the children were able to receive their shots because there were too many children.

Similarly, several participants were concerned that people from neighboring communities would discover the schedule for the mobile health clinics and travel to receive treatment, thus creating an additional burden on the clinics.

One participant felt that communication was integral to success of the mobile health clinic.

Mobile health clinics should have a nurse who knows the local language, so they can communicate with the people. This is very important.

A provider emphasized the importance of mobile health clinics being adequately stocked with supplies:

Mobile health clinics must be equipped with sufficient stock of materials—HIV test kits, TB testing, all the supplies—this is very important.

## DISCUSSION

Community members in rural Mozambique thought the most common health problems they faced (in order of frequency reported) were malaria, HIV, diarrhea, and TB. They indicated that the most common barriers to accessing health care were transportation costs, long distances, lack of transportation, and poor health facility services. Mobile health clinics present an opportunity to directly address these barriers by improving access to quality health care while decreasing the cost and time associated with accessing health services.

Stigma often remains an influencing factor on the decision to access HIV-related care in sub-Saharan Africa.^6^ By offering a broad range of clinical services, mobile health clinics can minimize stigma often associated with receiving care at HIV-specific facilities. In addition, educational outreach provided through mobile health clinics is a key component of both stigma reduction and HIV and TB awareness.

Participants identified concurrent use of the national health system and of the traditional health system for routine care. Physicians and nurses in the national health system often disapprove of the care provided by traditional healers, leading to conflict between physicians and nurses, healers, and patients.^17^^,^^18^ However, patients in this study indicated that traditional healers always referred them to the national health system for HIV care, suggesting an opportunity for collaboration between the 2 systems. For example, traditional healers could be used to provide health information and information on health care access with the arrival of mobile health clinics.

Traditional healers always referred patients to the national health system for HIV care, suggesting opportunities for collaboration.

A positive finding from the interviews is that all participants were accepting of mobile health clinics in their communities, potentially an indicator that community members will use the mobile health clinics. Use of mobile clinics has been successful elsewhere,^12^^,^^13^ and they have been shown to attract a greater proportion of first-time HIV testers than home-based and clinic-based testing.^19^^–^^21^ Prior mobile health clinic experience in underserved areas of South Africa with high HIV prevalence showed that such clinics can help facilitate earlier HIV diagnosis, supporting the World Health Organization recommendation for initiation of ART at lower CD4 thresholds.^22^

Most of the research thus far has been on using mobile clinics to improve access to and use of HIV testing and counseling services. For example, research has shown mobile health clinics to be a cost-effective approach for expanding HIV testing and counseling and in reaching persons for referral to treatment and care. A few studies have assessed use of mobile health clinics in Zambia to deliver ART and report improved anti-tuberculosis treatment outcomes and uptake and retention of ART.^24^^,^^25^ However, one potential limitation that must be accounted for is fuel, including availability of funding to purchase fuel to avoid visit delays or deviations from planned community visit days. A study in 3 African countries found that transportation cost and time and limited clinical hours are barriers to accessing health care.^23^ By visiting the community on a regular schedule, mobile health clinics have the potential to address these barriers, and hopefully reduce the financial cost of accessing health care, leaving more money to meet other necessary household expenses.[Fig f01]

**Figure f01:**
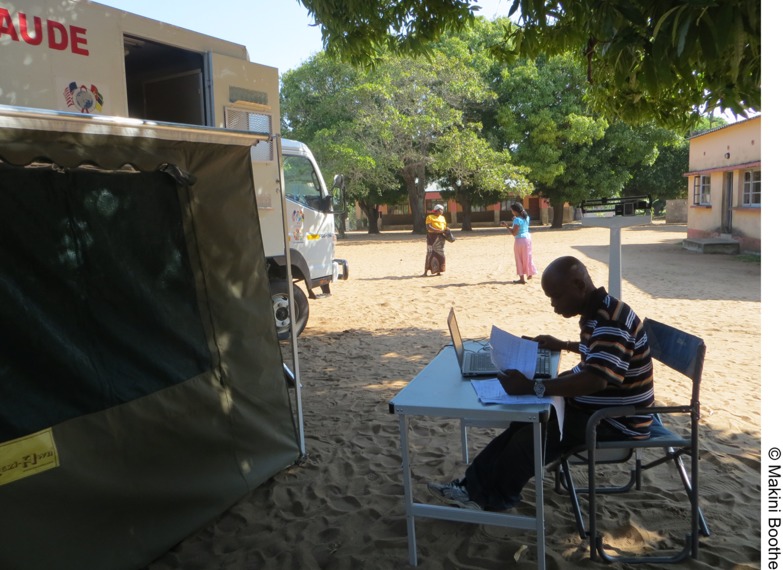
The driver of a mobile health clinic in Gaza province, Mozambique, serves a dual role for data entry during mobile health clinic visits.

Through the Mozambique National Health Plan, the Government of the Republic of Mozambique and the MOH have called for a national health policy focused on strengthening primary health care, improving infrastructure, and increasing community-level engagement with an initial focus on Gaza, Sofala, and Zambezia provinces.^4^ Our study suggests that mobile health clinics, working in collaboration with community health workers, can help strengthen selected primary care services, while increasing coverage of ART and improving retention of patients on ART over the long term. During multiple debrief sessions, results from this study were shared with partners to facilitate implementation of mobile health clinics in Gaza and Zamezia provinces.^26^ Major challenges to mobile health clinics implementation will include financing, human resources, long-term sustainability, and maintaining community acceptance. Coordination with community stakeholders such as community leaders and traditional healers is necessary for successful implementation of mobile health clinics.

### Study Limitations

Our study has several potential limitations, including the possibility of selection bias. National health system patients interviewed were already accessing health services, and traditional healers referred their own patients for interviews. This may have limited the range of perceptions gathered in our study, as persons not accessing the national or traditional health systems may have other opinions about mobile health clinics or on barriers to accessing care. Desirability bias could have led to participants giving answers that they thought would be viewed more favorably by the research team, and their answers may have been motivated by their desire to receive mobile health clinics or improved access to health care. As with all qualitative research, the generalizability of the findings is limited. However, the interviews were conducted in locations where mobile health clinics were planned for implementation.

## CONCLUSION

Mozambique's mobile health clinic program should help address some of the major barriers to uptake of HIV services, such as distance and lack of transportation to health facilities. Involvement of community leaders, providers, traditional healers, and patients and regularly scheduled visits are critical to the successful implementation of mobile health clinics.

## Notes

**Peer Reviewed**
